# Comprehensive microarray-based analysis for stage-specific larval camouflage pattern-associated genes in the swallowtail butterfly, *Papilio xuthus*

**DOI:** 10.1186/1741-7007-10-46

**Published:** 2012-05-31

**Authors:** Ryo Futahashi, Hiroko Shirataki, Takanori Narita, Kazuei Mita, Haruhiko Fujiwara

**Affiliations:** 1 Department of Integrated Biosciences, Graduate School of Frontier Sciences, University of Tokyo, Kashiwa, Chiba 277-8562, Japan; 2 Bioproduction Research Institute, National Institute of Advanced Industrial Science and Technology, Tsukuba, Ibaraki 305-8566, Japan; 3 National Institute of Genetics, Yata 1111, Mishima, Shizuoka 411-8540, Japan; 4 Nihon University College of Bioresource Sciences, Fujisawa, Kanagawa 252-0880, Japan; 5 National Institute of Agrobiological Sciences, Owashi, Tsukuba, Ibaraki 305-8634, Japan

**Keywords:** Butterfly, color pattern evolution, expressed sequence tag (EST), larval body marking, microarray, *Papilio polytes*, *Papilio xuthus*

## Abstract

**Background:**

Body coloration is an ecologically important trait that is often involved in prey-predator interactions through mimicry and crypsis. Although this subject has attracted the interest of biologists and the general public, our scientific knowledge on the subject remains fragmentary. In the caterpillar of the swallowtail butterfly *Papilio xuthus*, spectacular changes in the color pattern are observed; the insect mimics bird droppings (mimetic pattern) as a young larva, and switches to a green camouflage coloration (cryptic pattern) in the final instar. Despite the wide variety and significance of larval color patterns, few studies have been conducted at a molecular level compared with the number of studies on adult butterfly wing patterns.

**Results:**

To obtain a catalog of genes involved in larval mimetic and cryptic pattern formation, we constructed expressed sequence tag (EST) libraries of larval epidermis for *P. xuthus*, and *P. polytes *that contained 20,736 and 5,376 clones, respectively, representing one of the largest collections available in butterflies. A comparison with silkworm epidermal EST information revealed the high expression of putative blue and yellow pigment-binding proteins in *Papilio *species. We also designed a microarray from the EST dataset information, analyzed more than five stages each for six markings, and confirmed spatial expression patterns by whole-mount *in situ *hybridization. Hence, we succeeded in elucidating many novel marking-specific genes for mimetic and cryptic pattern formation, including pigment-binding protein genes, the melanin-associated gene *yellow-h3*, the ecdysteroid synthesis enzyme gene *3-dehydroecdysone 3b-reductase*, and *Papilio*-specific genes. We also found many cuticular protein genes with marking specificity that may be associated with the unique surface nanostructure of the markings. Furthermore, we identified two transcription factors, *spalt *and ecdysteroid signal-related *E75*, as genes expressed in larval eyespot markings. This finding suggests that *E75 *is a strong candidate mediator of the hormone-dependent coordination of larval pattern formation.

**Conclusions:**

This study is one of the most comprehensive molecular analyses of complicated morphological features, and it will serve as a new resource for studying insect mimetic and cryptic pattern formation in general. The wide variety of marking-associated genes (both regulatory and structural genes) identified by our screening indicates that a similar strategy will be effective for understanding other complex traits.

## Background

Body coloration is an ecologically important trait that is often involved in prey-predator interactions through mimicry and crypsis (camouflage) [1]. Because butterflies and moths spend most of their lives as larvae, which have soft bodies, they have developed a wide range of mechanisms to protect themselves from predators such as birds. The larval body markings of butterflies differ completely between closely related species and between individuals of the same species in different life stages [2,3]. Despite the variety and significance of the larval color patterns, few studies have been conducted at a molecular level compared with the available research on adult butterfly wing patterns [4-9].

Spectacular changes in color pattern are observed in the caterpillar of the swallowtail butterfly *Papilio xuthus*. As a young larva, it mimics bird droppings (mimetic pattern), and during the final molting period, it switches to a green camouflage coloration (cryptic pattern). In addition, larvae in the final instar stage have a large eyespot on their thoracic segment that is believed to be useful for avoiding predation. One of the main factors involved in larval pattern formation is insect hormones. Previously, we revealed that larval pattern switch is regulated by juvenile hormone (JH) [10]. The decline of JH titers on the first day of the fourth instar stage was the important factor controlling the formation of a green cryptic pattern in the fifth instar stage. The pattern transition occurs through ecdysis, and ecdysteroids appear to regulate the expression of several pigmentation genes. Topical application of 20-hydroxyecdysone alters the expression timing of several pigmentation genes [11,12]. Comparing gene expression between stages and markings represents a promising strategy for identifying the genes responsible for larval pattern formation.

Previously, we found several pigmentation genes using a cDNA subtraction and candidate gene approach [10,11,13-16]. Six melanin synthesis (or associated) genes, *tyrosine hydroxylase *(*TH*), *dopa decarboxylase *(*DDC*), *yellow*, *tan*, *laccase2 *and *guanosine triphosphate cyclohydrolase I *(*GTP-CH I*) are highly expressed in the presumptive black regions and *ebony *is highly expressed in the presumptive red region [16]. We also reported that the combination of *bilin-binding protein 1 (BBP1*) and *yellow-related gene *(*YRG*) correlated perfectly with larval blue, yellow, and green coloration in three *Papilio *species [12,17]. However, it was difficult to obtain novel marking-specific genes expressed at particular stages or genes with relatively low expression. For butterfly adult wing patterns, expressed sequence tag (EST) construction [18-20] and microarray analysis [6,21] have been conducted to obtain marking-associated genes. Microarray analysis revealed that the expression of both a patterning gene (transcription factor gene *optix*) and effector gene (ommochrome synthesis gene *cinnabar*) are associated with red wing patterns in *Heliconius *species [6,21].

In this study, we constructed an EST library of larval epidermis with more than 20,000 clones, and designed a microarray with the aim of comprehensively revealing the molecular mechanisms of larval mimetic and cryptic pattern formation. We performed microarray-based screening for marking-specific genes using six markings at 11 different stages, and verified the marking specificity of candidate genes by whole-mount *in situ *hybridization. We identified many novel marking-specific genes, including novel blue and yellow pigment-binding protein genes; a novel *yellow *family gene, the expression of which prefigures black cuticular markings; cuticular protein genes associated with marking-specific cuticular nanostructures; marking-associated regulatory genes; marking-specific ecdysteroid synthesis pathway genes; and *Papilio*-specific marking-associated genes. The data presented in this study provide a new resource to understand insect mimetic and cryptic pattern formation.

## Results and discussion

### Construction of a *Papilio *expressed sequence tag database

To comprehensively search the genes involved in larval body pattern formation, we constructed cDNA libraries derived from the whole epidermis during the third and the fourth molting periods, and sequenced 20,736 clones of *P. xuthus *and 5,376 clones of *P. polytes *(Table 1). We classified 4,114 and 1,614 nonredundant EST clusters and singletons from *P. xuthus *and *P. polytes*, respectively (Table 1 and see Additional files 1, 2, 3 and 4). From these, 773 and 178 clusters were considered isoforms or premature forms of other clusters (Additional files 3 and 4). Excluding these clusters, we identified 3,341 and 1,436 putative gene clusters (Additional files 1 and 2) for *P. xuthus *and *P. polytes*, respectively [DDBJ: AK401027-AK405767]. We assigned a serial number to each cluster in descending order of clone numbers (Additional files 1 and 2). We also determined full-length cDNA sequences for the 150 most highly expressed genes of *P. xuthus *through random amplification of cDNA ends technique. Among 3,341 gene clusters of *P. xuthus*, 2,746 genes had ORFs encoding predicted proteins longer than 50 amino acids, of which 2,276 had significant sequence similarities to monarch butterfly *Danaus plexippus *proteins [22], 2,189 to silkworm *Bombyx mori *proteins [23], and 1,957 to fruit fly *Drosophila melanogaster *proteins (Flybase ver. 5.42) (Table 2 and Additional file 5; cutoff threshold E values: *P *< 1e^-10 ^by BLASTP search). Based on the SignalP 3.0 program [24], we identified 625 genes encoding putative signal peptides (sequences are indicated in Additional file 1).

**Table 1:** Summary of *P. xuthus*, *P. polytes *and *B. mori *expressed sequence tag data sets

Species	*Papilio xuthus*	*Papilio polytes*	*Bombyx mori*^a^
Sequenced clone numbers (EST numbers)	20,736	5,376	10,368
	(36,589)	(8,549)	(6,653)
Number of clones corresponding to ribosomal RNA	693	30	automatically removed
Number of clones corresponding to mitochondrial DNA	217	24	automatically removed
Number of putative genes (including isoforms)	3,341	1,436	1,380
	(4,114)^b^	(1,614)^b^	(1,451)^b^

^a^Reported in [25]; ^b^773 clusters of *P. xuthus*, 178 clusters of *P. polytes*, and 71 clusters of *B. mori *were considered to be isoforms/premature forms of other clusters. EST: expressed sequence tags.

**Table 2:** Summary of *P. xuthus *gene set

Category	Numbers
*P. xuthus *genes (total)	3,341
*P. xuthus *genes with coding sequences (> 50 amino acids)	2,746
with homology to *Danaus plexippus *proteins (*P *< 1e^-10^)	2,276
with homology to *Bombyx mori *proteins (*P *< 1e^-10^)	2,189
with homology to *Drosophila melanogaster *proteins (*P *< 1e^-10^)	1,957

### Comparison of highly expressed genes among two *Papilio *species and *Bombyx*

**Figure 1:**
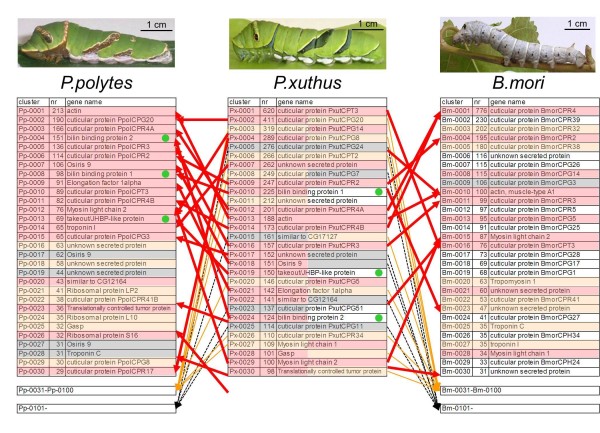
**Comparison of the 30 most highly expressed genes between *P. xuthus*, *P. polytes *and *B. mori***. *B. mori *data is from [25]. The 30 most highly expressed genes were color-coded in accordance with similarity of abundance of epidermal expressed sequence tag clones (red indicates genes which are among the 30 most highly expressed genes of both *P. xuthus *and *P. polytes*, or *P. xuthus *and *B. mori*; orange indicates genes which are among the 30 most highly expressed genes in one library and the 100 most highly expressed genes in another library; gray indicates the genes which are among the 30 most highly expressed genes in one species and out of the 100 most highly expressed genes in other species; and white indicates genes among the 30 most highly expressed genes in one species but not found in another species). Green circles indicate the *Papilio*-specific highly expressed genes ranking in the 30 most highly expressed genes of both *P. xuthus *and *P. polytes *datasets but not found in the *B. mori *library.

We previously reported a full-length EST database of larval epidermis in the fourth molting period of the silkworm *B. mori *[25]. Because *P. xuthus *and *P. polytes *larvae have a completely different appearance from *B. mori *larvae, there is a possibility that the combination of highly expressed genes may be different between *Papilio *and *Bombyx*. We color-coded the 30 most highly expressed genes in accordance with the similarity of the abundance of EST clones (Figure 1). The 30 most highly expressed genes of *P. xuthus *and *P. polytes *included many cuticular protein family genes, similar to *B. mori*. While the details of the 30 most highly expressed genes of *P. xuthus *were very similar to those of *P. polytes *(for example, 63% of the 30 most highly expressed genes were shared by *P. xuthus *and *P. polytes*, as indicated by the red shading in Figure 1), the combination of highly expressed genes were different from those of *B. mori *(Figure 1), which may reflect the differences of the larval appearance between *Papilio *and *Bombyx*. For example, three genes, *Px-0010 *(*Pp-0008*), *Px-0019 *(*Pp-0013*) and *Px-0024 *(*Pp-0004*), were included among the 30 most highly expressed genes in both *P. xuthus *and *P. polytes *(indicated by green circles in Figure 1), but homologs of these genes were not found in the *B. mori *epidermal EST database. Several cuticular protein genes were only found in *B. mori*, and many cuticular protein genes ranking high in *Papilio *species were not among the 100 most highly expressed genes in *B. mori *(Figure 1). Although the accuracy of expression levels was unclear based only on the clone numbers of the EST database, the relatively high expression of the 30 most highly expressed genes of *P. xuthus *was subsequently confirmed by microarray analysis. Notably, *Px-0010 *and *Px-0024 *had sequence similarity with bilin-binding protein (BBP), and *Px-0019 *had sequence similarity with takeout/JH-binding protein (JHBP), which we will discuss in detail in subsequent sections.

### Microarray-based screening for marking-specific genes in *P. xuthus*

**Figure 2:**
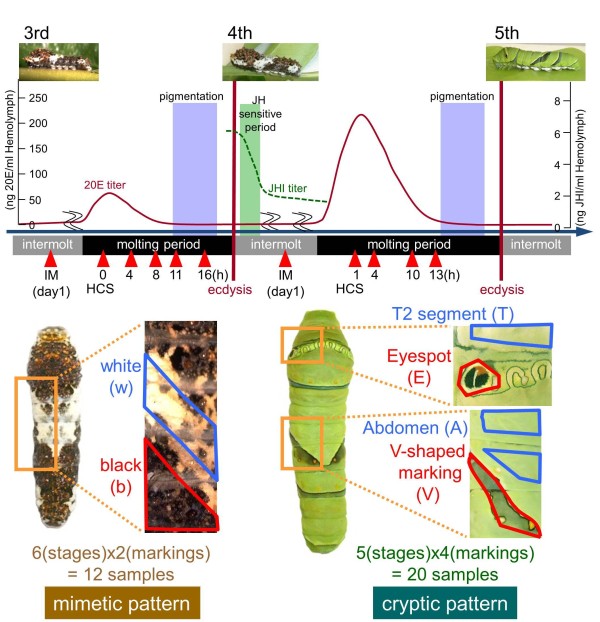
**Scheme of microarray analyses**. Larval epidermis was dissected at 11 different stages indicated by red arrowheads. The epidermis was separated for each marking as indicated below here: A: abdominal segments with no markings of cryptic pattern; b: black region of mimetic pattern; E: eyespot of cryptic pattern; T: thoracic segments with no markings of cryptic pattern; V: V-shaped markings of cryptic pattern w: white region of mimetic pattern. Ecdysteroid and juvenile hormone titers in hemolymph are based on the previous reports [10,11]. The cryptic pattern is assumed to be determined in the juvenile hormone sensitive period (green box) [10]. The grey box indicates the cuticular pigmentation stage [12,13]. HCS: head capsule slippage; IM: intermolt.

One advantage of the *Papilio *larva is its sufficiently large size for separating each marking by dissection, that is, these larvae are convenient for performing microarray analysis of the markings. In addition to the EST dataset, to construct the *Papilio *microarray we also independently cloned 77 genes known to be associated with wing-marking patterns or ecdysteroid signal cascade (including *Distal-less*, *cinnabar*, and *ecdysone receptor*) [21,26-30] by using degenerate primers (Additional file 6). We used 32 samples in the microarray (six stages and two markings for mimetic pattern and five stages and four markings for cryptic pattern, Figure 2).

To determine whether we could compare microarray data across all samples, we first compared the normalized signal intensity of housekeeping genes (ribosomal protein genes). We found that these genes were ubiquitously expressed as expected, and their normalized signal intensity among the samples was within two-fold of their average values in most cases (Additional files 7 and 8). We also checked several melanin synthesis genes, for which we have previously reported their stage-specific and marking-specific expressions, and confirmed the expected specific expression (see below). Thus, we considered that a comparison of the normalized signal intensity across all of the examined samples is reliable for screening of the novel marking- and stage-specific genes.

**Figure 3:**
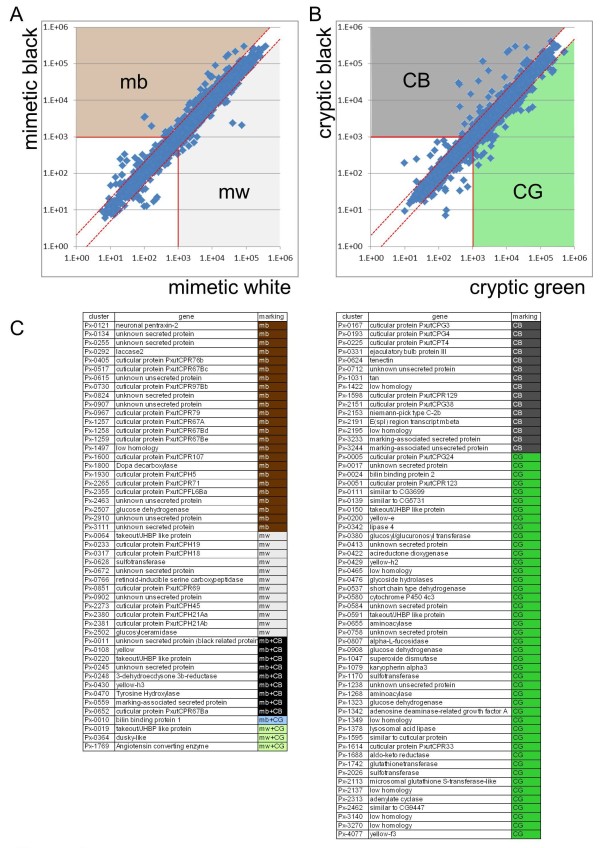
**Screening for marking-specific genes**. **(A) **Scatter plots of normalized signal intensity of mimetic white (*x *axis, average of six stages) versus mimetic black (*y axis*, average of six stages). Genes with average intensity more than two-fold (red dashed lines) between mimetic black and mimetic white, and also with an average signal intensity higher than 1,000 (red solid lines) were categorized as marking-specific genes. **(B) **Scatter plots of normalized signal intensity of cryptic green (*x *axis, average of five stages) versus cryptic black (*y axis*, average of five stages). For cryptic markings, T and A were regarded as cryptic green, and E and V were regarded as cryptic black (see Figure 2). **(C) **List of marking-specific genes. CB: cryptic black-enriched; CG: cryptic green-enriched; mb: mimetic black-enriched; mw: mimetic white-enriched.

To screen for marking-specific genes, we compared the average normalized signal intensities between mimetic white (mw) and mimetic black (mb) samples, and between cryptic green (CG) (thoracic 2 segment and abdomen region with no markings) and cryptic black (CB) samples (eyespot and V-shaped markings). We categorized genes with an average signal intensity more than two-fold change between mb and mw, and/or CG and CB, and an average signal intensity higher than 1,000 as marking-specific genes. Using these criteria, we regarded 34 genes as mb-, 13 as mw-, 24 as CB- and 48 as CG-enriched genes (Figure 3). Among the marking-specific genes, nine genes (*Px-0011*, *Px-0108*, *Px-0220*, *Px-0245*, *Px-0248*, *Px-0430*, *Px-0470*, *Px-0559 *and *Px-0652*) were regarded as both mb- and CG-enriched genes. This group contained melanin synthesis and associated genes, the takeout/JHBP gene family, cuticular protein genes, the 3-dehydroecdysone 3b-reductase (3DE 3b-reductase) gene and genes of unknown function. In contrast, three genes (*Px-0019*, *Px-0364 *and *Px-1769*) were regarded as both mw- and CG-enriched genes. *Px-0019 *was a *Papilio*-specific highly expressed gene based on a comparison of the 30 most highly expressed genes (indicated by green circles in Figure 1). One gene, *Px-0010*, already reported as *BBP1*, was regarded as mb- and CG-enriched gene. This is reasonable because *BBP1 *expression was detected in blue spots within the black region of the mimetic pattern and the green region of the cryptic pattern [12,15].

**Figure 4:**
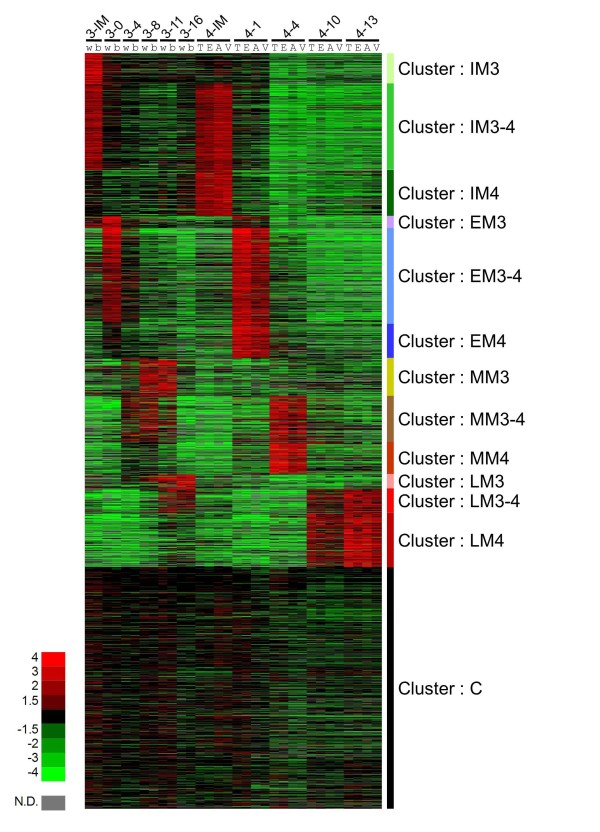
**Heat map of the relative expression level of 3,376 *P. xuthus *genes by microarray results**. Red and green indicate higher and lower expression level, respectively. The stage-specific co-expression clusters are shown right. C: constantly expressed genes; IM3: genes enriched in the intermolt of the third instar; IM3-4: genes enriched in the intermolt of both the third and the fourth instar; IM4: genes enriched in the intermolt of the fourth instar; EM3: genes enriched in the early stage of the third molt; EM3-4: genes enriched in the early stage of both the third and the fourth molts; EM4: genes enriched in the early stage of the fourth molt; MM3: genes enriched in the middle stage of the third molt; MM3-4: genes enriched in the middle stage of both the third and the fourth molts; MM4: genes enriched in the middle stage of the fourth molt; LM3: genes enriched in the late stage of the third molt; LM3-4: genes enriched in the late stage of both the third and the fourth molts; LM4: genes enriched in the late stage of the fourth molt. Stage and marking of each sample is shown above (for example, 4-10E, eyespot at 10 hours after head capsule slippage of the fourth molting period). Red indicates positive values and green indicates negative values (color spectrum bar is shown left; N.D., not detected). See also Figure 2.

To screen for instar- and stage-specific genes, expression profiles were grouped by self-organizing maps with GenePattern software [31], which is often used to summarize microarray data. By self-organizing maps analysis, expression profiles were grouped into 13 co-expression clusters (Figure 4). The largest cluster (cluster C) contained approximately one third of the genes with constant expression profiles (for example, housekeeping genes such as ribosomal protein genes). Other clusters contained intermolt-enriched genes (IM3, IM3-4 and IM4) and genes enriched in the early (EM3, EM3-4 and EM4), middle (MM3, MM3-4 and MM4), and late stages of molting (LM3, LM3-4 and LM4). Genes belonging to clusters other than cluster C were expected to be regulated by ecdysteroid because their expression levels differed in the molting period when the ecdysteroid titer changes (see Figure 2). The third instar- (IM3, EM3, MM3 and LM3) and fourth instar-enriched genes (IM4, EM4, MM4 and LM4) were expected to be positively and negatively regulated by JH, respectively. The genes with similar expression patterns between the third and fourth instars (IM3-4, EM3-4, MM3-4, LM3-4 and C) were assumed to be JH-independent genes. The marking specificity and instar and/or stage specificity for each gene is indicated in Additional file 1.

### Identification of several candidates for blue and yellow pigment-binding proteins

**Figure 5:**
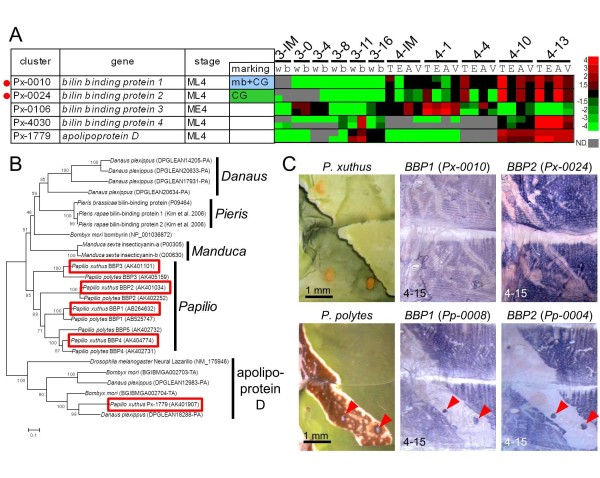
**Expression pattern of bilin-binding protein genes**. **(A) **Heat map of four bilin-binding protein genes and their related gene apolipoprotein D in *P. xuthus*. Red indicates positive values and green indicates negative values (color spectrum bar is shown to the right; N.D., not detected). Red circles indicate the genes examined by whole-mount *in situ *hybridization in (C). Stage and marking of each sample is shown above. The stage-specific co-expression cluster and marking specificity of each gene are also shown. See also Figures 2 to 4. **(B) **Neighbor joining tree of bilin-binding protein and its related genes based on their amino acid sequences. The numbers at the tree nodes represent the bootstrap values. The scale bars indicate the evolutionary distance between the groups. Accession numbers or gene model numbers are shown in parenthesis. Red boxes indicate the *P. xuthus *genes. Predicted genes of *D. plexippus *and *B. mori *were from MonarchBase http://monarchbase.umassmed.edu/ and KAIKObase http://sgp.dna.affrc.go.jp/KAIKObase/, respectively. **(C) **Spatial expression patterns of two bilin-binding protein gene mRNAs in *P. xuthus *and *P. polytes *larvae during the fourth molt. Numbers in each panel indicate molt stage-hours after head capsule slippage (HCS). Red arrowheads indicate blue spot region of *P. polytes*.

One of the most obvious characteristics of the *Papilio *larval cryptic pattern is the overall green coloration. Lepidopteran larval green coloration consists of blue bile pigment (bilin) and yellow carotenoids in general [32]. These pigments usually exist as pigment-protein complexes *in vivo *[33]. We previously reported that the combination of *BBP1 *and *YRG *correlated perfectly with larval blue, yellow and green coloration in three *Papilio *species [12]. Although the high expression of *BBP1 *(*Px-0010*) in the CG region was indicated by the EST database, microarray analyses and whole-mount *in situ *hybridization (Figure 5A, C), *YRG *(*Px-0080*) expression was relatively low (Additional file 7). This imbalance in the expression level of putative blue and yellow pigment-binding proteins suggested the presence of other pigment-binding proteins. No gene homologous to the carotenoid-binding protein reported from the silkworm *B. mori *[34] was found in the *Papilio *EST datasets. Instead, *Px-0019 *(related to the takeout/JHBP gene family) and *Px-0024 *(related to the lipocalin family), both among the 30 most highly expressed genes in the EST library, appeared to be good candidates for carotenoid-binding protein and BBP genes (see Figure 1). Both *Px-0019 *and *Px-0024 *were CG-enriched in the microarray-based gene expression analysis (Figure 3, Additional file 9). The expression profile of *Px-0024 *was very similar to that of *BBP1 *(*Px-0010*) (Figure 5A). Molecular phylogeny indicated that *Px-0024 *and its *P. polytes *ortholog *Pp0004 *were members of the lipocalin family, a putative binding protein for lipophilic substances, including the BBP of *Pieris rapae *and insecticyanin (BBP) of *Manduca sexta *(Figure 5B). Thus, we named *Px-0024 *(and its ortholog *Pp0004*), *BBP2*. Molecular phylogeny also indicated that these genes had lineage-specific gene duplication, and *P. xuthus *and *P. polytes *had at least four and five paralogous genes, respectively. Almost identical marking-specific expression between *BBP1 *and *BBP2 *in both *P. xuthus *and *P. polytes *was indicated by microarray analysis and whole-mount *in situ *hybridization (Figure 5C), suggesting that these two genes may function coordinately as blue pigment-binding proteins. As it is supposed that BBPs of the saturniid silkworm *Rhodinia fugax *exist as a dimer in epidermis [35], it is possible that *BBP1 *and *BBP2 *form heterodimers. Notably, bombyrin of *B. mori *is not associated with blue coloration, and molecular phylogeny indicated that the blue pigment-binding proteins of *Pieris*, *Manduca *and *Papilio *do not form a single cluster, suggesting that blue pigment-binding proteins evolved independently to serve a common physiological role in each organism.

**Figure 6:**
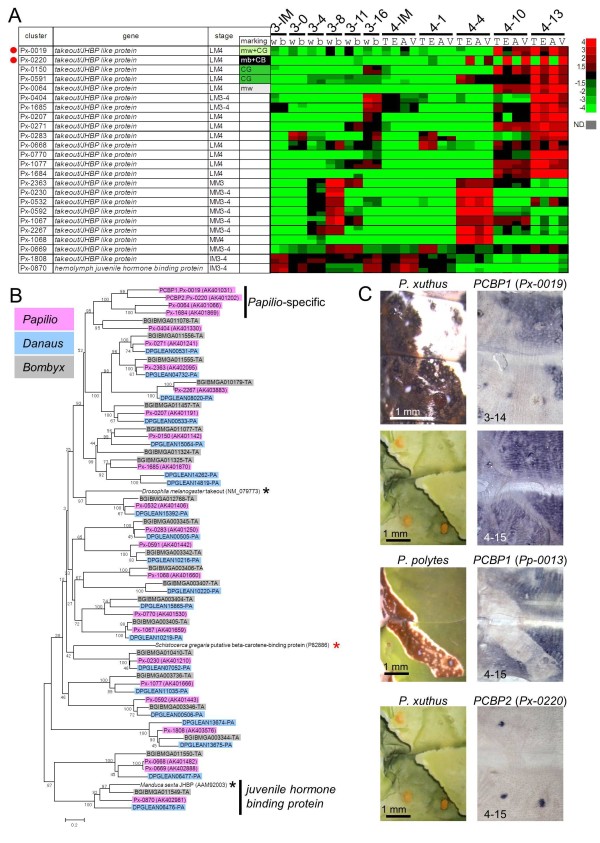
**Expression pattern of putative carotenoid-binding protein genes**. **(A) **Heat map of 24 *takeout/juvenile hormone*-*binding protein *(*JHBP*) family genes in *P. xuthus*. Red indicates positive values and green indicates negative values (color spectrum bar is shown to the right; N.D., not detected). Red circles indicate the genes examined by whole-mount *in situ *hybridization in (C). Stage and marking of each sample is shown above. The stage-specific co-expression cluster and marking specificity of each gene are also shown. See also Figures 2 to 4. **(B) **Neighbor joining tree of *takeout/JHBP *family genes based on their amino acid sequences. The numbers at the tree nodes represent the bootstrap values. The scale bars indicate the evolutionary distance between the groups. *P. xuthus*, *D. plexippus *and *B. mori *genes are shaded in red, blue and gray, respectively. Red asterisk indicates carotenoid-binding protein of locust *Schistocerca gregaria*, and black asterisks indicate takeout protein of *Drosophila melanogaster *and JHBP of *Manduca sexta*. Accession numbers are shown in parenthesis. Predicted genes of *D. plexippus *and *B. mori *were from MonarchBase http://monarchbase.umassmed.edu/ and KAIKObase http://sgp.dna.affrc.go.jp/KAIKObase/, respectively. **(C) **Spatial expression patterns of two putative carotenoid-binding protein mRNAs in *P. xuthus *and *P. polytes *larvae during the fourth and the third molts. Numbers in each panel indicate molt stage-hours after head capsule slippage. JHBP: juvenile hormone-binding protein.

Regarding *Px-0019*, its spatial expression pattern associated with the cryptic pattern was similar to those of *BBP1 *and *BBP2 *in *P. xuthus *(Figure 6C). Similar to the expression pattern of *YRG *reported previously [12], the expression of *Pp-0013 *(the ortholog of *Px-0019*) in *P. polytes *was not detected in the blue spot region (Figure 6C) whereas *BBP1 *and *BBP2 *expression was clearly detected (red arrowheads in Figure 5C). Molecular phylogeny indicated that *Px-0019 *formed a large cluster consisting of the takeout/JHBP gene family and 23 other genes in the *P. xuthus *EST database (Figure 6B). Notably, this cluster included the carotenoid-binding protein of the locust *Schistocerca gregaria *(red asterisk in Figure 6B) [36], suggesting that *Px-0019 *is one of the *P. xuthus *carotenoid-binding proteins. *Px-0019 *formed a *Papilio*-specific cluster with three other *Papilio *genes (indicated by '*Papilio*-specific' in Figure 6B). Among the 24 *Papilio *takeout/JHBP genes, *Px-0019 *and its closest homolog gene *Px-0220 *had the strongest marking specificity in the microarray expression profile (Figure 6A). In contrast to that of *Px-0019*, the expression level of *Px-0220 *was higher in eye spot regions and V-shaped markings (Figure 6A). Consistent with the microarray results, the spatial expression pattern of *Px-0220 *perfectly correlated with the yellow spot region of the V-shaped markings (Figure 6C). The expression patterns of *Px-0019 *and *Px-0220 *strongly suggest that these *Papilio*-specific family genes bind yellow carotenoid pigments with different spatial regulation. We therefore named these genes putative carotenoid-binding proteins (PCBP1 and PCBP2, respectively). Moderate PCBP1 (*Px-0019*) expression was also detected in the white region of the mimetic pattern by microarray analysis and *in situ *hybridization, suggesting that this gene also colocalizes with white pteridine/uric acid pigments (Figure 6A, C).

Because JHBP has high ligand specificity [37,38] and it has been assumed to be monomeric in solution [39], PCBP1 and PCBP2 may bind to different types of carotenoids. Other takeout/JHBP genes (for example, *Px-0150 *and *Px-0591*) also had marking specificity and were enriched in the fourth instar (Figure 6A), suggesting that these genes also have a supporting role in carotenoid binding. Because the presence of several carotenoids, including alpha-carotene, beta-carotene and lutein, have been reported for *P. xuthus *[40], these takeout/JHBP family genes of *P. xuthus *may each recognize a different carotenoid.

Notably, the locust carotenoid-binding protein (red asterisk in Figure 6B) did not form a single cluster with *Px-0019 *or *Px-0220*, which is similar to the case of *BBP*, suggesting that both blue and yellow pigment-binding proteins evolved convergently among insect species. Green coloration among lepidopteran larvae appears to have emerged independently in the phylogenetic tree [2]. The independent occurrence of BBPs and carotenoid-binding proteins within the lipocalin family genes and takeout/JHBP family genes may reflect the convergent evolution of larval green coloration.

### Identification of the novel marking-specific melanin synthesis gene, *yellow-h3*

**Figure 7:**
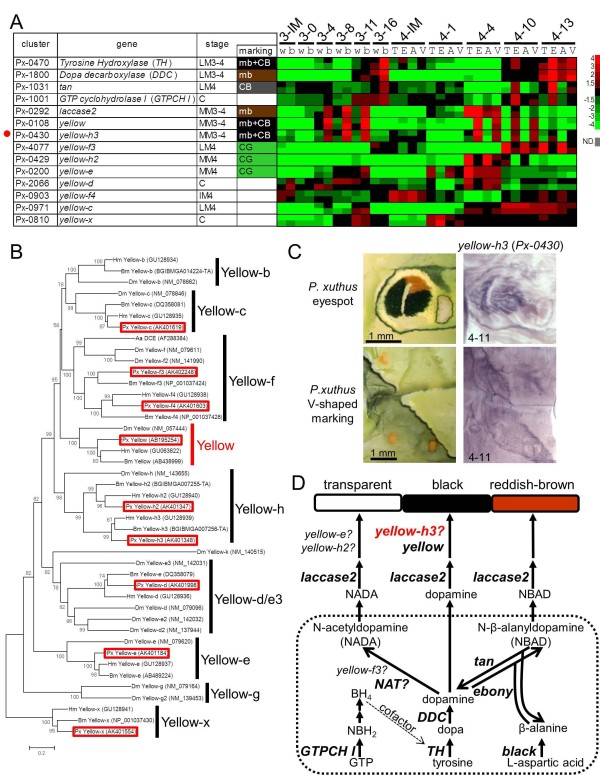
**Spatial expression pattern of melanin synthesis genes**. **(A) **Heat map of the relative expression level of melanin synthesis/related genes and *yellow *family genes in *P. xuthus*. Red indicates positive values and green indicates negative values (color spectrum bar is shown to the right; N.D., not detected). Red circle indicates the gene examined by whole-mount *in situ *hybridization in (C). Stage and marking of each sample is shown above. The stage-specific co-expression cluster and marking specificity of each gene are also shown. See also Figures 2 to 4. **(B) **Neighbor joining tree of *yellow *family genes based on their amino acid sequences. The numbers at the tree nodes represent the bootstrap values. The scale bars indicate the evolutionary distance between the groups. Accession numbers are shown in parenthesis. Red boxes indicate the *P. xuthus *genes. Yellow-g gene has not been reported in lepidopteran species [43]. **(C) **Spatial expression patterns of *yellow-h3 *mRNA in *P. xuthus *larva during the fourth molt. Numbers in each panel indicate molt stage-hours after head capsule slippage. **(D) **Biosynthetic pathway underlying the formation of melanin. Dopamine, N-beta-alanyldopamine (NBAD) and N-acetyldopamine (NADA)are used in the production of Dopamine-melanin (black and brown), NBAD pigment (yellow or reddish brown), and NADA pigment (transparent) in insects [5,16,45,62]. Aa DCE: *Aedes aegypti *dopachrome conversion enzyme; BH_4_: tetrahydrobiopterin; Bm: *Bombyx mori*; DDC: dopa decarboxylase; Dm: *Drosophila melanogaster; *GTP: guanosine triphosphate; GTP-CH I: GTP cyclohydrolase I; Hm: *Heliconius melpomene*; NADA: N-acetyldopamine; NAT: arylalkylalamine-N-acetyltransferase; NBAD: N-beta-alanyldopamine; NBH_2_: dihydroneopterin triphosphate; Px: *Papilio xuthus*. TH: tyrosine hydroxylase.

We previously reported that several melanin-related genes were associated with stage- and species-specific black larval cuticular markings in three *Papilio *species [10-14,16]. The microarray results were consistent with our previous findings. Both *TH *(*Px-0470*) and *DDC *(*Px-1800*) were strongly expressed in the black markings during the latter half of the molting period; *yellow *(*Px-0108*) and *laccase2 *(*Px-0292*) were strongly expressed in the black markings during the middle of the molting period; *tan *(*Px-1031*) was strongly expressed in the cryptic black markings at the latter half of the molting period; and *GTP-CH I *(*Px-1001*) was strongly expressed in the black markings only in specific stages (Figure 7A). In addition to these genes, Yellow-f-related genes have been reported as dopachrome-converting enzymes for melanin biosynthesis (for example, *D. melanogaster yellow-f *and *yellow-f2 *and the *Aedes aegypti *dopachrome-converting enzyme, Figure 7B) [41,42]. In *B. mori *and *D. melanogaster*, 10 and 14 *yellow *family genes (which have major royal jelly protein motifs) have been reported (Figure 7B) [40,42]. We obtained all *yellow *family genes in *P. xuthus *(red boxes in Figure 7B) excluding *yellow-b*. In lepidopteran species, *yellow-f3 *and *yellow-f4 *are the closest homologs to *Drosophila yellow-f/yellow-f2 *(Figure 7B) [43].

Unexpectedly, *yellow-f3 *(*Px-4077*) and *yellow-f4 *(*Px-0903*) of *P. xuthus *were not upregulated in black markings, but instead were CG-enriched (Figure 7A). Conversely, *yellow-h3 *(*Px-0430*) was upregulated in black markings, and it exhibited a very similar expression pattern to that of *yellow *(Figure 7A). Using whole-mount *in situ *hybridization, we confirmed that the spatial expression pattern of *yellow-h3 *prefigured the black markings similar to *yellow *(Figure 7C). The only previous report concerning the role of *yellow-h3 *in coloration was the demonstration of its association with black regions in *Heliconius *wings by RT-PCR, but its spatial expression patterns have not been clarified [43]. Our data, combined with the *Heliconius *results, suggest that *yellow-h3 *functions in melanin biosynthesis in lepidopteran species (Figure 7D) similarly to *yellow-f *and *yellow-f2 *in *Drosophila*.

Based on our microarray analysis, three *yellow *family genes, *yellow-f3*, *yellow-h2 *and *yellow-e*, were regarded as CG-enriched genes (Figures 3 and 7A). *yellow-f3 *was highly expressed during the late molting period similar to *TH *and *DDC*, and *yellow-h2 *and *yellow-e *were highly expressed during the middle of the molting period similar to *yellow *(Figure 7A), implying that these genes may function in inhibiting melanin pigmentation. In the silkworm *B. mori*, *yellow-e *disruption promoted melanin pigmentation in the larval head and tail, where strong *yellow-e *expression was detected [44], which is consistent with our results. In melanin synthesis, it is assumed that arylalkylamine-N-acetyltransferase (NAT) activity is involved in synthesis of N-acetyldopamine, a precursor of colorless cuticle [5,45,46]. However, disruption of *NAT *in *B. mori *resulted in an overall blackish phenotype only in the adult stage, whereas it had little effect on larval pigmentation [46,47]. Our EST datasets did not contain *NAT *or *NAT*-like genes, suggesting that *NAT *gene is not the primary factor for colorless cuticular production in larval stages. Because green is the color of the epidermis seen through the colorless cuticle, the observation of CG-enriched *yellow *family genes (CG in Figure 3) implies that *yellow *family genes are major negative regulators of melanin pigmentation in the larval stage instead of the *NAT *gene (Figure 7D). Taxa-specific gene duplication found between *Drosophila *and lepidopteran species (for example, *yellow-f*, *yellow-h*, Figure 7B), and the reverse function implicated for *yellow-f *family genes between Diptera and Lepidoptera suggest that the function of the *yellow *gene family has diversified among insect taxa.

### Marking-specific cuticular protein genes

**Figure 8:**
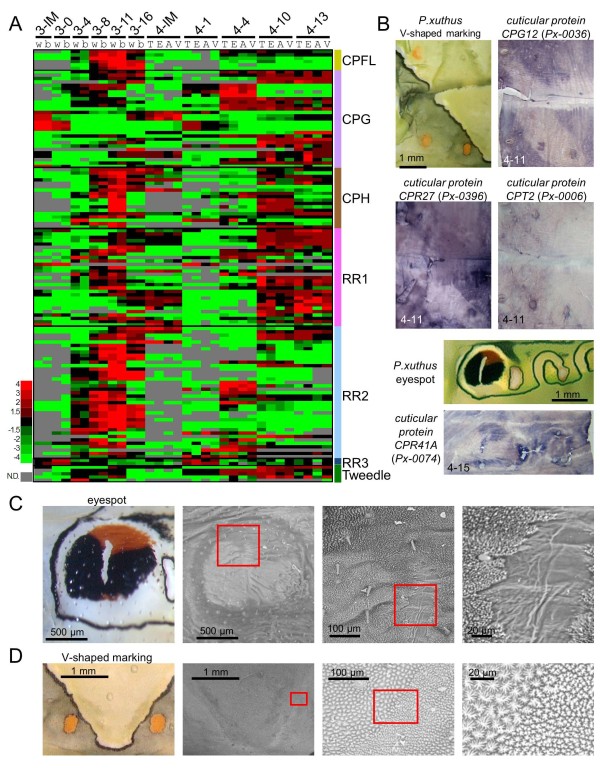
**Marking-specific expression of cuticular protein genes and marking-associated cuticular nanostructure**. **(A) **Heat map of the relative expression level of 128 cuticular protein genes. Red indicates positive values and green indicates negative values (color spectrum bar is shown to the left; N.D., not detected). Stage and marking of each sample is shown above. Cuticular protein genes were categorized by the motif indicated in the right. See also Figure 2. **(B) **Spatial expression patterns of cuticular protein genes' mRNA in *P. xuthus *larva during the fourth molt. Numbers in each panel indicate molt stage-hours after head capsule slippage. **(C) **Cuticular nanostructure (scanning electron microscopy image) of eyespot region. Magnified view of red square regions is shown to the right. **(D) **Cuticular nanostructure (scanning electron microscopy image) of V-shaped marking region. Magnified view of red square regions is shown to the right.

The 30 most highly expressed genes of the *Papilio *epidermal EST dataset included many cuticular protein genes similar to *B. mori *(Figure 1) [25]. In *B. mori*, more than 200 cuticular protein genes have been reported [48,49]. The heat map of 128 cuticular protein genes of *P. xuthus *indicated the clear stage specificity of most of the cuticular protein genes (Figure 8A). Cuticular protein genes can be divided into several groups based on amino acid sequence similarity [48,50]. Many genes with a CPFL or RR2 motif were highly expressed in the third instar (mimetic pattern) although stage specificity was not completely associated with this motif, as we reported previously [15]. Interestingly, many cuticular protein genes also displayed marking specificity (Figure 3 and Additional file 7), which was confirmed by *in situ *hybridization (Figure 8B). The cuticular proteins *CPG12 *(glycine-rich motif, *Px-0036*) and *CPR27 *(RR-1 motif, *Px-0396*) were highly expressed in the CG region as well as the yellow spot region in V-shaped markings (Figure 8B), and *CPT2 *(Tweedle motif, *Px-0006*) was highly expressed in the yellow spot region as well as the boundary of the V-shaped region (Figure 8B). Strong expression of *CPR41A *(RR1 motif, *Px-0074*) was detected in the red regions of the eyespot (Figure 8B). To examine whether the different spatial expression of the cuticular protein genes affected exoskeletal structures, we examined the cuticular surface by electron microscopy. We found that cuticular structures were different at each marking (Figure 8C, D), and we could easily recognize the eyespot region through electron microscopy (Figure 8C). The surface nanostructure was fine in the black region of the eyespot and coarse in the yellowish green region around the eyespot. The surface nanostructure of the red region was intermediate between the black and green regions. The white stripe center of the eyespot had a very smooth surface. We previously reported that muscle was attached to this white stripe region [14]. The surface nanostructure dramatically changed at the boundary of the V-shaped markings (Figure 8D). The surface nanostructure of the black stripe region was fine and the green region was coarse similar to the eyespot. These results indicated that color pattern and surface structure are tightly related, which is similar to the adult wing scale in which clear correlations between color and structure have been reported [51,52]. Although the precise role of each cuticular protein remains unclear, specific cuticular proteins may have the function of transporting or maintaining the specific cuticular pigments.

### Candidates for marking-associated patterning genes

**Figure 9:**
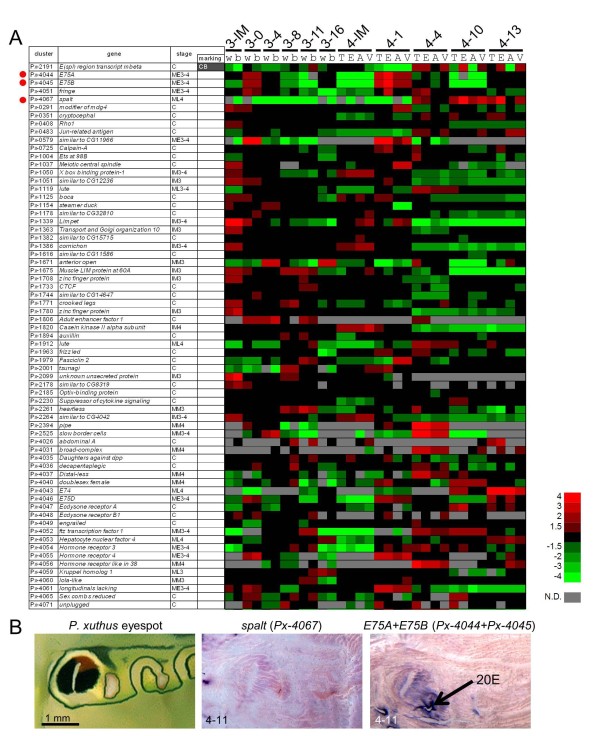
**Expression pattern of candidates for patterning genes**. **(A) **Heat map of the relative expression level of 67 regulatory genes (transcription factors and signal transducers). Red indicates positive values and green indicates negative values (color spectrum bar is shown to the right; N.D., not detected). Red circles indicate the genes examined by whole-mount *in situ *hybridization in (B). Stage and marking of each sample is shown above. The stage-specific co-expression cluster and marking specificity of each gene are also shown. See also Figures 2 to 4. **(B) **Spatial expression patterns of *spalt *and *E75 *(probe designed at the common region of *E75A *and *E75B*) mRNA in *P. xuthus *larva during the fourth molt. Numbers in each panel indicate molt stage-hours after head capsule slippage. Black arrows indicate the position of 20E application.

Although several regulatory genes have been reported for butterfly adult wing pattern formation [6,7,26,29], it is still unclear which gene controls the larval body markings. We surveyed the marking specificity of 76 regulatory genes such as transcription factors and signal transducers. According to microarray analysis, nine genes (*trithorax*, *polybromo*, *aristaless*, *cut*, *doublesex *(male type), *optomotor blind*, *ultrabithorax*, *vein *and *wingless*) were expressed at lower levels than measureable, and most genes had no clear marking specificity (Figure 9A). However, we found that *E(spl) region transcript mbeta *(*Px-2191*) had clear marking specificity (CB in Figure 3) and four other genes *E75A*, *E75B*, *fringe *and *spalt*, also exhibited higher expression in the eyespots based on the heat map (Figure 9A). We confirmed the stronger expression of *spalt *in the eyespot by whole-mount *in situ *hybridization (Figure 9B) although we could not detect the positive signals of the four other genes, perhaps because of the low signal intensity in the examined stage (whole-mount *in situ *hybridization is applicable after the middle stage, 11 hours after head capsule slippage (HCS), of the molting period when cuticular apolysis is complete). Notably, *spalt *expression coincided with the black markings of several butterfly wing patterns [7,29]. Taken together, *spalt *appeared to be a positive regulator of melanin pigmentation in both larvae and adult butterflies.

*E75A *and *E75B *are involved in the ecdysteroid signaling cascade, and their expression is induced by ecdysteroids [30]. We therefore examined the effect of ecdysteroids by the topical application method [11] and found that higher *E75 *expression is maintained in black eyespot regions (Figure 9B; probes were designed to target common regions of *E75A *and *E75B*). The expression pattern of *E75 *coincided with the eyespot pattern, which was similar to *yellow *[11], suggesting that *E75 *had a marking specificity in the early molting stage. In *Manduca sexta*, both *E75A *and *E75B *are involved in the stage-specific gene expression of *DDC *both directly and indirectly [30]. Our results suggest that *E75A *and/or *E75B *regulate both the marking specificity and stage specificity of several black marking-associated genes. As we described previously, several cuticular protein genes also exhibited marking specificity and stage specificity (Figure 8). Although it is still not clear how color and nanostructure are determined, one possible explanation is that marking-specific transcription factors involved in the ecdysteroid signaling cascade regulate both pigmentation and cuticular protein genes. *E75 *is one strong candidate mediator of the hormone-dependent coordination of larval pattern and nanostructure formation.

Although *spalt *and *E75 *were the only two transcription factor genes for which we were able to detect marking-associated expression by whole-mount *in situ *hybridization, microarray analysis suggested that *E(spl) region transcript mbeta *and *fringe*, both involved in the Notch signaling pathway, also have marking specificity. In the butterfly wing pattern, *Notch *is involved in intervein markings [53], and *fringe *is upregulated by 20-hydroxyecdysone [54]. The Notch signaling pathway may also be involved in hormone-dependent pattern formation in butterfly larvae.

### The marking-specific ecdysteroid biosynthesis enzyme gene, *3-dehydroecdysone 3b-reductase*

**Figure 10:**
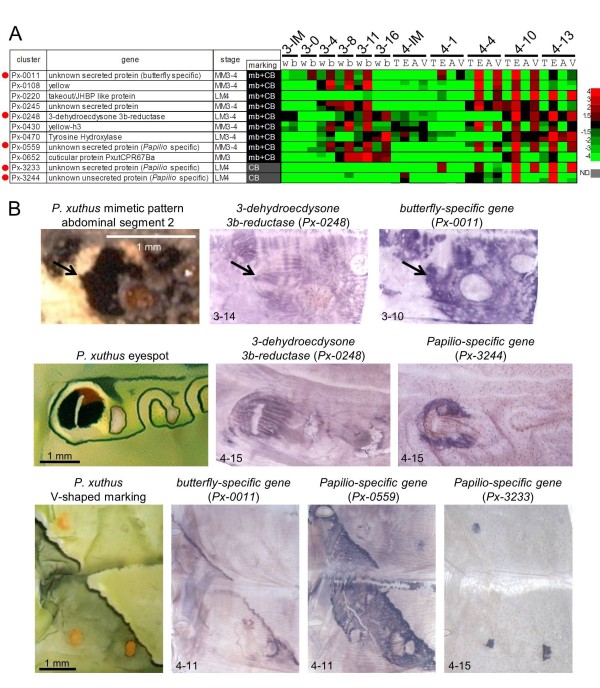
**Expression pattern of novel marking-specific genes**. **(A) **Heat map of the relative expression level of nine marking-specific genes regarded as mimetic black and cryptic black, and two *Papilio*-specific genes (*Px-3233*, *Px-3244*) regarded as cryptic black. Red indicates positive values and green indicates negative values (color spectrum bar is shown to the right; N.D., not detected). Red circles indicate the genes examined by whole-mount *in situ *hybridization in (B). Stage and marking of each sample is shown above. The stage-specific co-expression cluster and marking specificity of each gene are also shown. See also Figures 2 to 4. **(B) **Spatial expression patterns of novel marking-specific genes' mRNA in *P. xuthus *larva during the third and fourth molts. Numbers in each panel indicate molt stage-hours after head capsule slippage. Arrow indicates black spot region on the abdominal segment 2 of mimetic pattern.

Unexpectedly, we found that the *3DE 3b-reductase *gene (*Px-0248*), which converts inactivated 3-dehydroecdysone to ecdysone [55,56], had clear marking specificity during the late stage of the molting period. According to whole-mount *in situ *hybridization findings, *3DE 3b-reductase *was highly expressed in black markings in both mimetic and cryptic patterns (Figure 10), which is very similar to the *TH *and *DDC *expression patterns. The simultaneous expression of *3DE 3b-reductase *and melanin synthesis enzyme genes suggested that localized regulation of ecdysteroid titers in the epidermis is also involved in marking-specific gene regulation. Relatively high concentrations of ecdysone in the black markings may be important for regulating the *TH *and *DDC *expressions during the pigmentation stages.

### *Papilio*-specific marking-associated genes

Among 2,746 *P. xuthus *genes (ORF > 50 amino acids), more than 400 genes had no sequence similarity with the genome sequences of other insects, including silkworm and monarch butterfly (Additional file 5). Through microarray analysis and whole-mount *in situ *hybridization, we found three *Papilio*-specific genes (that is, has no sequence similarity with known proteins including the monarch butterfly *Danaus*, *Bombyx *genome sequences, and the Butterfly Base sequence database [57] with clear marking specificity (*Px-0559*, *Px-3233 *and *Px-3244*), and one gene, *Px-0011*, with only low sequence similarity with the monarch butterfly (that is, butterfly specific). *Px-0559*, *Px-3233 *and *Px-3244 *expression coincided with black, yellow and eyespot markings, respectively (Figure 10). *Px-0011 *expression was also strongly associated with black markings (Figure 10). These proteins have no known domains and display no similarity to any characterized proteins. *Px-0011 *and *Px-3233 *have signal peptide sequences, and *Px-0011*, *Px-0559 *and *Px-3233 *are threonine-, arginine-, and tyrosine-rich proteins, respectively (Additional file 1). These genes were expressed during the middle or late stages of the molting period, suggesting that these genes were structural proteins. Although the precise function of these genes is currently unknown, this is the first example of species-specific genes associated with pattern formation.

## Conclusion

In this study, we succeeded in obtaining a large-scale catalog of marking-specific genes and stage-specific genes from the swallowtail butterfly larvae by microarray analysis based on EST datasets. We confirmed the marking specificity for transcription factors, hormone-related genes, cuticular protein genes, pigment synthesis and binding genes, and novel *Papilio*-specific genes by whole-mount *in situ *hybridization. The marking-specific genes identified in this study indicated that the molecular mechanisms of insect pigmentation are likely to be both conserved and diversified across insect taxa. For example, several of the melanin synthesis genes with clear marking specificity in *Papilio *species (Figure 7) [12,16] have been reported to be responsible for the larval color mutants of the silkworm *B. mori *[16,58,59], as well as associated with the cuticular pigmentation of various insect orders [5,45,60-62], which suggests their conserved role in cuticular pigmentation across insect taxa. Conversely, our results also indicate that genes that have several paralogs in one species tend to diversify their function across insect taxa, such as *yellow *family genes, lipocalin family genes and takeout/JHBP family genes (Figures 5, 6, 7). We also found that there were several *Papilio*-specific genes with clear marking specificity, suggesting that species-specific genes contribute to marking formation. Notably, some of the regulatory genes involved in larval pattern appeared to be different from those of adults (for example, *Distal-less *involvement in eyespots was less obvious in larvae), whereas others appeared to participate in both larval and adult pattern formation (for example, *spalt *expression associated with black markings). Furthermore, we found that both the transcription factor *E75 *and the ecdysteroid synthesis enzyme *3DE 3b-reductase *had clear marking specificity, suggesting their involvement in ecdysteroid-dependent coordinated gene regulation in larval pattern formation. Ecdysteroid is also involved in butterfly adult wing pattern [28], which suggests the possibility that these genes contribute to pattern formation in the adult wing. The wide variety of marking-associated genes identified by our screening indicates that the strategy utilized in this study is effective for clarifying pattern formation in species without genome information. The gene collection and the expression profile presented in this study will be invaluable for exploring not only *Papilio *but also insects in general.

## Methods

### Experimental animals and developmental staging

*P. xuthus *was either purchased from Eiko-Kagaku (Osaka, Japan), kindly provided by Dr. Akira Yamanaka (Yamaguchi University, Japan), or collected from the field. Larvae were reared on leaves of *Zanthoxylum ailanthoides *(Rutaceae) at 25°C under long-day conditions (16 h light and 8 h dark). The staging of the molting period was based on the time when HCS occurred, as well as spiracle and hair pigmentation [12].

### Construction and sequencing of the cDNA library

Total epidermal RNAs of *P. xuthus *and *P. polytes *were isolated from 50 individuals per species during the molting period (35 individuals at third molt and 15 individuals at fourth molt) using TRI reagent (Sigma-Aldrich, St. Louis, MO, USA) according to the manufacturer's instructions. After the attached muscle and fat body were removed, whole dorsal integuments from the thoracic 2 segment to the abdominal 7 segment were dissected from larvae. Total RNAs were mixed and subjected to cDNA library construction.

The cDNA library construction was carried out by the Dragon Genomics Center (Takara Bio Inc., Mie, Japan) by the following procedure. The mRNA was purified using an Oligotex-dT30 Super mRNA purification Kit (Takara, Bio Inc., Mie, Japan), and then cDNA libraries were constructed using a cDNA synthesis Kit (Stratagene, La Jolla, CA, USA). First-strand cDNA synthesis was carried out with oligo d(T)_18 _primers. Synthesized cDNA were size-selected and ligated into the *EcoR*I and *Xho*I sites of pBluescript II SK(+) vector (Stratagene). The ligated products were then transformed into competent DH10B *Escherichia coli *cells by electroporation. The transformed cells were plated onto Luria Broth media and incubated overnight.

For sequencing of cDNA libraries, white colonies were randomly selected and sequenced from both ends using an ABI prism 3130 Genetic Analyzer (Applied Biosystems, Foster City, CA, USA), which was carried out at National Institute of Genetics (Shizuoka, Japan) with the support of the MEXT Genome Support Project for the *P. xuthus *cDNA library, and at the National Institute of Agrobiological Sciences (Ibaraki, Japan) for the *P. polytes *cDNA library. All EST sequences have been deposited [DDBJ: FY174038-FY210626, FY302525-FY358875]. Among these ESTs, sequences corresponding to ribosomal RNA [DDBJ: AB674749] and mitochondrial DNA [DDBJ: EF621724] were eliminated. The remaining ESTs were subjected to cluster analysis using a Phred/Phrap/Consed software package [63]. After automatic clustering, we checked every sequence manually for each alignment, and divided and reassembled the putative chimeric sequences. SignalP 3.0 [24] was used for signal peptide prediction.

### Microarray experiments and data analysis

The Papilio 15 K oligo-microarray slide (60-mer oligonucleotides on 15,208 spots, Agilent Technologies, Palo Alto, CA, USA) was constructed using 8,000 *Papilio *unique sequences (3,376 from *P. xuthus*, 4,612 from *P. polytes *and 12 from *P. machaon*). We could not design an appropriate probe for 42 genes because of low sequence complexity (indicated by 'n.e.' in Additional file 1). Probe sequences were designed using Agilent's web portal eArray [64], and one or two probes each for *P. xuthus*, *P. machaon *and *P. polytes *gene were constructed (probe sequences of *P. xuthus *are shown in Additional file 7).

For the microarray experiment of mimetic pattern, total epidermal RNAs of six developmental stages (0, 4, 8, 11, 16 hours after HCS of the third molting period and intermolt (day 2) of the third instar) were isolated from white (abdominal 2 to 4 segments) and black (abdominal 4 and 5 segments) regions independently (see Figure 2, left). For the microarray experiment of cryptic pattern, total epidermal RNAs of five developmental stages (1, 4, 10, 13 hours after HCS of the fourth molting period and intermolt (day 2) of the fourth instar) were isolated from the thorax (thoracic 2 segment with no markings), eyespot (thoracic 3 segment), abdomen (abdominal 3 and 4 segments with no markings), and V-shaped marking (abdominal 4 and 5 segments) regions independently (see Figure 2, right).

We performed two color microarray hybridizations. Mimetic white, cryptic thorax and cryptic abdomen were independently labeled with cyanine 3-CTP (Cy3), and mimetic black, cryptic eyespot, and cryptic V-shaped markings were independently labeled with cyanine 5-CTP (Cy5) in all stages. Double-stranded cDNA and labeled cRNA were synthesized using the Low RNA Input Linear Amplification Kit according to the manufacturer's instructions (Agilent Technologies). Total RNA (400 ng each for Cy3 and Cy5) was converted to cDNAs with T7 promoter oligo-dT primer and Moloney Murine Leukemia Virus reverse transcriptase. Second-strand cDNA was transcribed to cRNA with T7 RNA polymerase and 10 mM Cy3 or Cy5. The labeled cRNA was purified using an RNeasy Mini Kit (Qiagen, Tokyo, Japan).

Hybridization was performed using an In situ Hybridization Kit Plus (Agilent Technologies). One microgram each of Cy3-labeled cRNA and Cy5-labeled cRNA were mixed, fragmented and hybridized to each oligo-microarray at 65°C for 17 h at 4 rpm. The arrays were washed in 6 × saline sodium citrate, 0.005% TritonX-102 for 10 min at room temperature and in 0.1 × saline sodium citrate, 0.005% TritonX-102 for 5 min at 4°C. Intensities of the hybridized probes were detected with an Agilent G2565BA Microarray Scanner with 10 mm scan resolution, and the signals were extracted with G2565AA Feature Extraction Software v.7.1 (Agilent Technologies). Normalization was performed 'per spot' and 'per chip' using the GENESPRING program. The low expression level spots in all cells were removed (fluorescence intensity is approximately lower than 100). Microarray data were deposited in the Gene Expression Omnibus [GEO:GSE37920]. The normalized fluorescence intensity of all samples and each probe sequence are shown in Additional file 7.

We used microarray-based screening to obtain the genes involved in the overall steps of pattern formation, with the following strategies: comparing gene expression between different markings to identify marking-specific genes; comparing gene expression between mimetic and cryptic patterns to identify instar-specific genes; and checking the gene expression profile during the molting periods to identify stage-specific genes. To do this, we dissected larval epidermis at 11 different stages (Figure 2, red arrowheads) for microarray screening. Because cryptic patterns are assumed to be determined in the JH-sensitive period (green box in Figure 2) [10], six different stages before the JH-sensitive period were expected to be associated with the mimetic pattern and five different stages after the JH-sensitive period were analyzed for genes associated with cryptic pattern. For the mimetic pattern, we separately dissected the white region (w) and the black region (b), whereas for the cryptic pattern, we separately dissected the thoracic 2 segment (T), eyespot (E), abdomen region with no markings (A) and V-shaped markings (V) (Figure 2). Expression profiles of 11 developmental stages or six markings (mimetic white, mimetic black, cryptic thorax, cryptic eyespot, cryptic abdomen and cryptic V-shaped marking) were grouped by self-organizing maps with GenePattern software [31]. This algorithm is often used to summarize microarray data. We obtained expression profiles in terms of relative expression rather than absolute expression levels by dividing the average expression levels of each gene.

### Phylogenetic analysis

Alignment was conducted based on translated protein sequences using Clustal W program implemented in MEGA5, and phylogenetic trees were constructed by the Neighbor joining method with the MEGA5 program [65]. The confidence of the various phylogenetic lineages was assessed by the bootstrap analysis.

### Whole-mount *in situ *hybridization

Whole dorsal integuments from the thoracic 2 segment to the abdominal 7 segment were dissected from larvae. After the body fat and muscle attached to the epidermis were carefully removed, the larval epidermis was fixed immediately in 4% paraformaldehyde in PBS (137 mM NaCl, 8.10 mM Na_2_HPO_4_, 2.68 mM KCl and 1.47 mM KH_2_PO_4_, pH 7.4). Whole-mount *in situ *hybridization was performed as described previously [13,15-17]. An RNA probe for each gene was prepared using the DIG RNA Labeling Kit (Roche Biochemicals, Mannheim, Germany). A color reaction for digoxigenin-labeled antisense RNA probes was performed at room temperature in 100 mM Tris-HCl, 100 mM NaCl and 50 mM MgCl_2 _(pH 9.5) containing 3.5 μL/mL 5-bromo-4-chloro-3-indolyl-phosphate, 4-toluidine salt and 4.5 μL/mL nitroblue tetrazolium chloride. Digoxigenin-labeled sense strand probes were used as negative controls.

### Scanning electron microscopy analysis

Cuticular nanostructure of eyespot and V-shaped marking region was observed by scanning electron microscopy using a Hitachi TM-1000 scanning electron microanalyzer (Hitachi High-Technologies Corp., Tokyo, Japan).

## Abbreviations

3DE 3b-reductase: 3-dehydroecdysone 3b-reductase; BBP: bilin-binding protein; CB: cryptic black; CG: cryptic green; Cy3: cyanine 3-CTP; Cy5: cyanine 5-CTP; *DDC*: *dopa decarboxylase*; EST: expressed sequence tag; *GTP-CH I*: *guanosine triphosphate cyclohydrolase I*; HCS: head capsule slippage; JH: juvenile hormone; JHBP: juvenile hormone-binding protein; mb: mimetic black; mw: mimetic white; NAT: arylalkylamine-N-acetyltransferase; ORF: open reading frame; PBS: phosphate-buffered saline; PCBP: putative carotenoid-binding protein; RT-PCR: reverse transcriptase polymerase chain reaction; *TH*: *tyrosine hydroxylase*; *YRG*: *yellow-related gene*.

## Competing interests

The authors declare that they have no competing interests.

## Authors' contributions

RF and HF designed the research. RF performed the manual assembly and annotation of EST datasets. RF and HS analyzed the EST database and generated *in situ *hybridization data. TN sequenced the cDNA library of *P. xuthus*, and KM sequenced the cDNA library of *P. polytes*. RF performed and analyzed the microarray data. RF prepared figures and RF and HF wrote the manuscript. All authors read and approved the final manuscript.

## Supplementary Material

Additional file 1 **List of 3,341 nonredundant *P. xuthus *genes**. cluster nr: indexing identity number for each cluster; Total ESTs: number of ESTs in each cluster; sequence: 'majority rule' consensus sequence (with N masking regions of low complexity); length: length of consensus sequence; member: list of all individual EST names in the cluster; peptide sequence: predicted peptide sequence; SignalP: putative signal peptide sequence predicted by SignalP 3.0 program; DmGene: best hit from BLASTP analysis to *Drosophila melanogaster *peptides (Flybase v. 5.42); DmCG: CG number of best hit from BLASTP analysis to *D. melanogaster *peptides; DmE-value: Evalue of best hit from BLASTP analysis to *D. melanogaster *peptides; BmGene: best hit from BLASTP analysis to *Bombyx mori *peptides; BmE-value: Evalue of best hit from BLASTP analysis to *B. mori *peptides; DpGene: best hit from BLASTP analysis to *Danaus plexippus *peptides; DpE-value: Evalue of best hit from BLASTP analysis to *D. plexippus *peptides; Remarks: gene name of each cluster; accession number: GenBank accession number; co-expression cluster: co-expression cluster by microarray analysis (n.d.: not detected: n.e.: not examined); marking specificity: average intensity of mimetic black (mb) vs. mimetic white (mw) and/or cryptic green (CG) vs. cryptic black (CB) over two-fold is indicated. Click here for file

Additional file 2 **List of 1,436 *P. polytes *nonredundant genes**. Explanation of column contents are the same as Additional file 1. Click here for file

Additional file 3 **List of identified putative isoforms or premature transcripts of *P. xuthus***. Click here for file

Additional file 4 **List of identified putative isoforms or premature transcripts of *P. polytes***. Click here for file

Additional file 5 **The Venn diagram of *P. xuthus *genes**. The numbers of homologous genes shared between *P. xuthus *epidermal expressed sequence tags and other insect genomes are shown (cutoff threshold E values: *P *< 1e^-10 ^by BLASTP search). Click here for file

Additional file 6 **List of 77 additionally cloned *P. xuthus *genes**. Explanation of column contents is the same as Additional file 1. Click here for file

Additional file 7 **Microarray result of *P. xuthus *genes**. Probe sequences, normalized signal intensity of each sample, sum of signal intensity, and relative expression level of each sample are shown. Click here for file

Additional file 8 **Heat map of the relative expression level of 78 ribosomal protein genes in *P. xuthus***. Red indicates positive values and green indicates negative values (color spectrum bar is shown to the right; N.D., not detected). Stage and marking of each sample is shown above. The sage-specific co-expression cluster and marking specificity of each gene are also shown. See also Figures 2 to 4. Click here for file

Additional file 9 **Heat map of the relative expression level of the 30 most highly expressed genes in *P. xuthus***. Stage and marking of each sample is shown above. Red indicates positive values and green indicates negative values (color spectrum bar is shown to the right; N.D., not detected). The stage-specific co-expression cluster and marking specificity of each gene are also shown. See also Figures 2 to 4. Click here for file
