# Supplementary figures and images for: Comprehensive microarray-based analysis for stage-specific larval camouflage pattern-associated genes in the swallowtail butterfly, Papilio xuthus
Source: BMC Biol. 2012 May 31;10:46. doi: 10.1186/1741-7007-10-46 (PMC3386895; doi:10.1186/1741-7007-10-46)

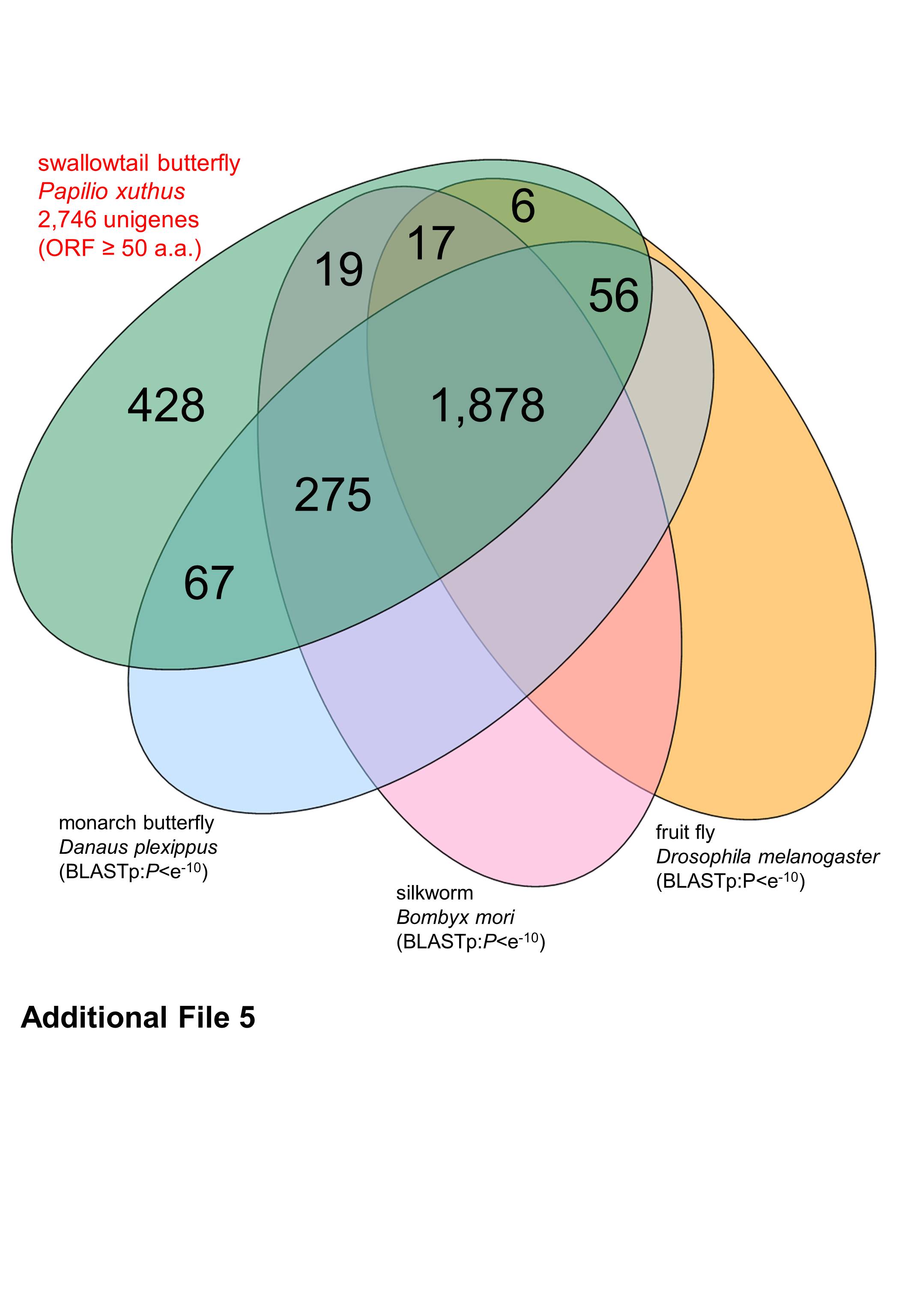

Supplement: Additional file 5 — The Venn diagram of P. xuthus genes. The numbers of homologous genes shared between P. xuthus epidermal expressed sequence tags and other insect genomes are shown (cutoff threshold E values: P < 1e-10 by BLASTP search). [file 1741-7007-10-46-S5.JPEG]

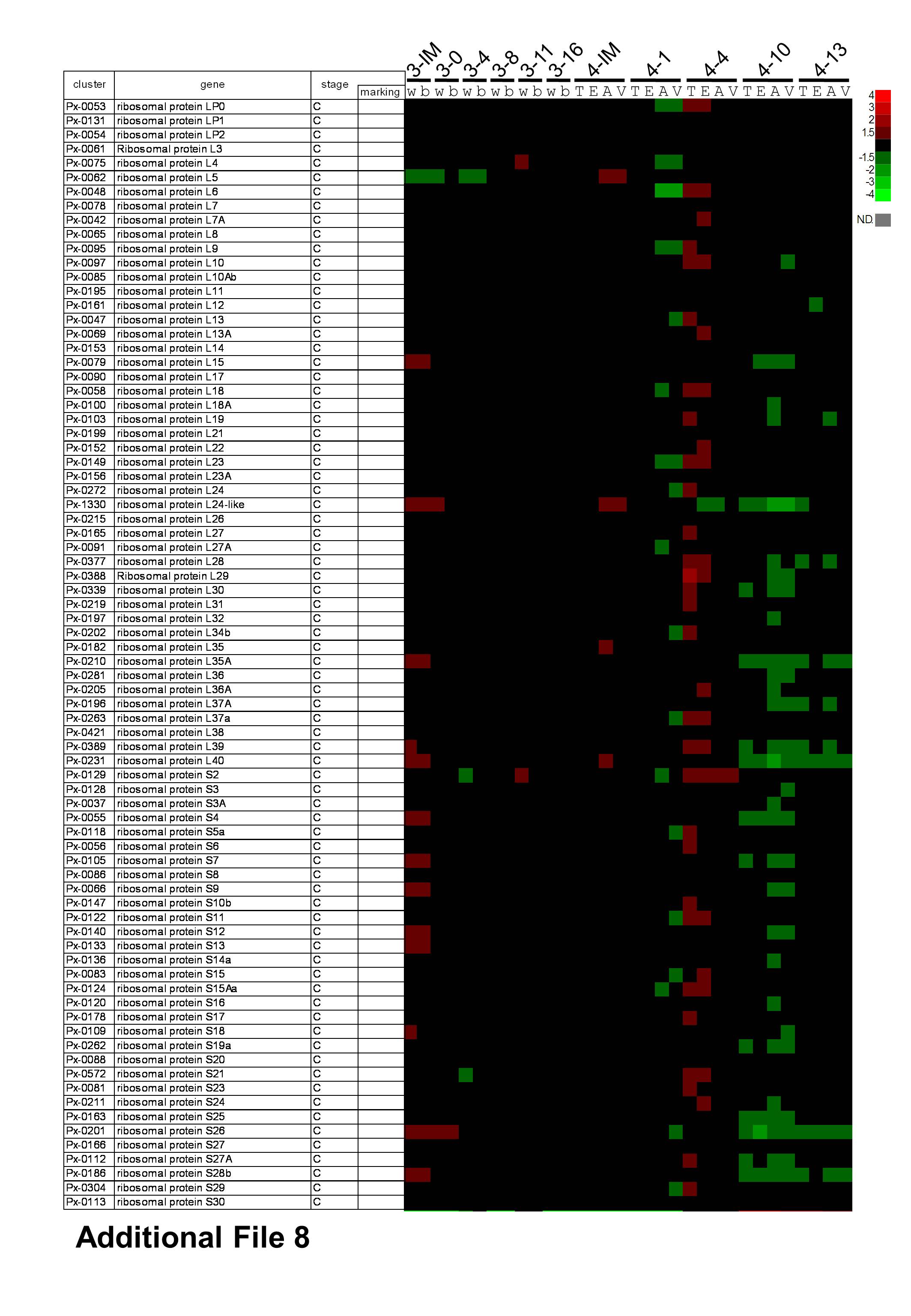

Supplement: Additional file 8 — Heat map of the relative expression level of 78 ribosomal protein genes in P. xuthus. Red indicates positive values and green indicates negative values (color spectrum bar is shown to the right; N.D., not detected). Stage and marking of each sample is shown above. The sage-specific co-expression cluster and marking specificity of each gene are also shown. See also Figures 2 to 4. [file 1741-7007-10-46-S8.JPEG]

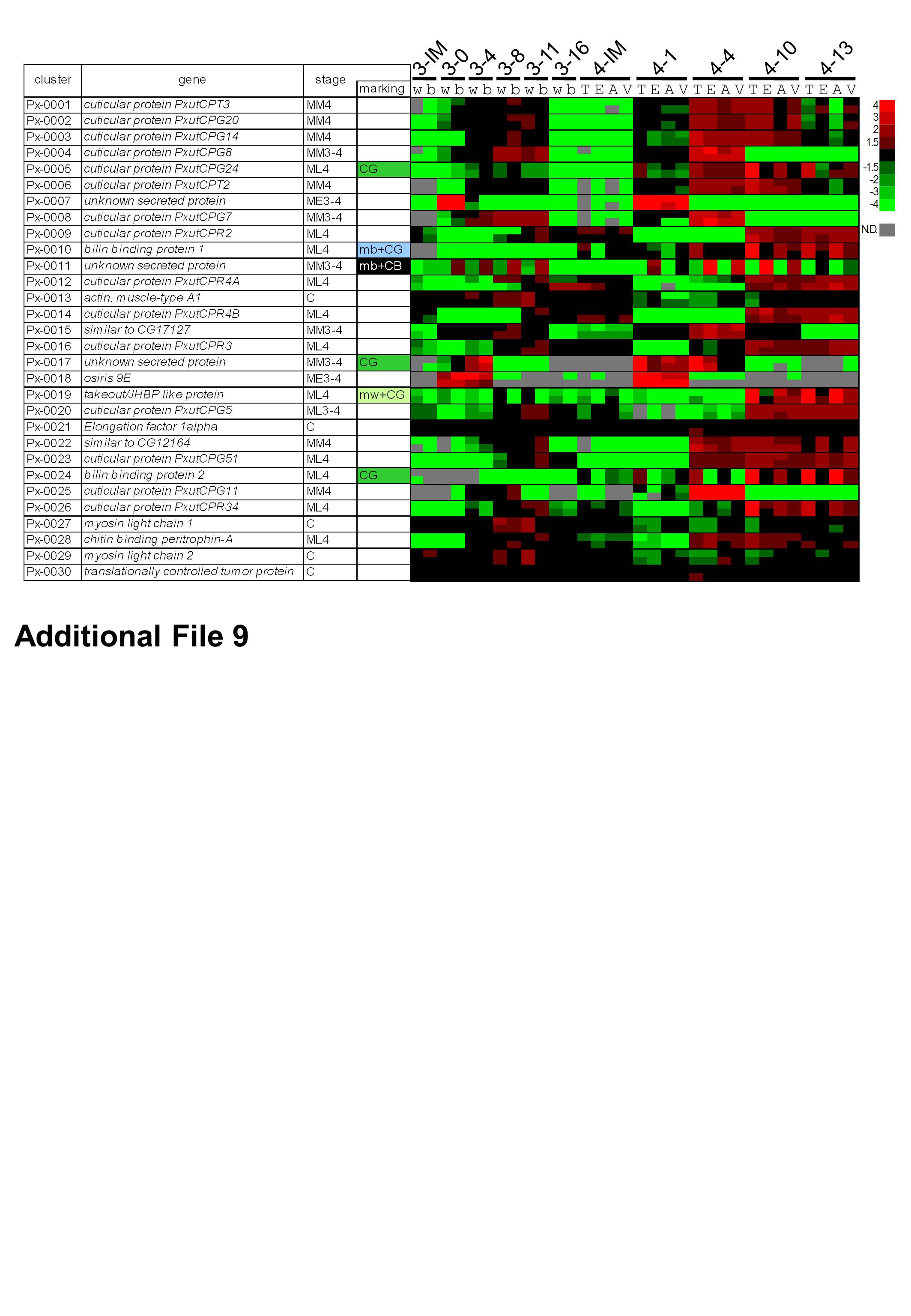

Supplement: Additional file 9 — Heat map of the relative expression level of the 30 most highly expressed genes in P. xuthus. Stage and marking of each sample is shown above. Red indicates positive values and green indicates negative values (color spectrum bar is shown to the right; N.D., not detected). The stage-specific co-expression cluster and marking specificity of each gene are also shown. See also Figures 2 to 4. [file 1741-7007-10-46-S9.JPEG]
